# Hydrogel-Nanoparticles Composite System for Controlled Drug Delivery

**DOI:** 10.3390/gels4030074

**Published:** 2018-09-04

**Authors:** Emanuele Mauri, Anna Negri, Erica Rebellato, Maurizio Masi, Giuseppe Perale, Filippo Rossi

**Affiliations:** 1 Department of Chemistry, Materials and Chemical Engineering “G. Natta”, Politecnico di Milano, via Mancinelli 7, 20131 Milan, Italy; emanuele.mauri@polimi.it (E.M.); anna1.negri@mail.polimi.it (A.N.); erica.rebellato@mail.polimi.it (E.R.); maurizio.masi@polimi.it (M.M.); 2 Biomaterials Laboratory, Institute for Mechanical Engineering and Materials Technology, SUPSI—University of Applied Sciences and Arts of Southern Switzerland, via Cantonale 2C, Galleria 2, 6928 Manno, Switzerland; giuseppe.perale@supsi.ch

**Keywords:** drug delivery, hydrogel, polymeric nanoparticles, release system

## Abstract

Biodegradable poly(ethylene glycol)-block-poly(-lactic acid) (PEG-*b*-PLA) nanoparticles (NPs) were prepared by nanoprecipitation with controlled dimension and with different electric charges, as monitored by dynamic light scattering (DLS). Then NPs were loaded within hydrogels (HG) developed for biomedical applications in the central nervous system, with different pore sizes (30 and 90 nm). The characteristics of the resulting composite hydrogel-NPs system were firstly studied in terms of ability to control the release of small steric hindrance drug mimetic. Then, diffusion-controlled release of different charged NPs from different entangled hydrogels was studied in vitro and correlated with NPs electric charges and hydrogel mean mesh size. These studies showed different trends, that depend on NPs superficial charge and HG mesh size. Release experiments and diffusion studies, then rationalized by mathematical modeling, allowed us to build different drug delivery devices that can satisfy different medical needs.

## 1. Introduction

Research in the area of controlled drug delivery systems (DDS) has gained increasing importance over the last 50 years, because of the advantages in terms of safety, efficacy, and patient convenience that these long-acting systems provide [1,2]. In particular, DDS can present many advantages, such as maintenance of drug concentration within therapeutic values for a sustained and prolonged period of time, reducing side effects and increasing patient’s compliance, due to the reduction of the number of administrations [3,4]. In the huge field of biomaterials, for this purpose, increased attention is given to polymers, not only to fabricate three-dimensional scaffolds, but also to develop injectable colloidal systems able to carry and release drugs [5,6]. In this framework, one of the most suitable classes of compounds is represented by hydrogels (HG) [7,8]. These polymers are typically soft and elastic due to their thermodynamic compatibility with water and can form a three-dimensional polymeric network able to carry cells and drugs. Indeed, HGs show many additive characteristics that make them excellent delivery vehicles [9,10,11]. For example mucoadhesive and bioadhesive characteristics allow remaining in situ, enhancing drug residence time and tissue permeability, being relatively deformable, elastic, and so able to conform to the shape to which they are confined [12,13].

Moreover, their high similarity with the native extracellular matrix could give them the opportunity of being used to serve as dual-purpose devices: (i) support material for cells during tissue regeneration, and (ii) DDS for drug payload [14,15,16]. However behind many advantages there are also disadvantages present. Among them, the most important ones are the incapacity to carry and release hydrophobic drugs, control the release of low steric hindrance molecules, and finalize cell selective therapies [12,17,18]. A class of materials able to overcome these disadvantages is represented by polymeric nanoparticles (NPs). These are gaining increasing interest due to their flexibility in terms of size, hydrophilic/hydrophobic tunable nature, and surface functionalization [19,20,21]. Moreover, they show high advantages in drug delivery by targeting molecules in specific cells and sustaining and tuning drug release over time. In previous work, we demonstrated that this could be a winning point [22,23,24]. However, this advantage could also represent a huge problem if the pharmacological therapy has to be directed to other cell lines or districts [25,26,27], or it is needed to maintain them localized within the target tissue [28]. This task is very problematic, mainly because of the presence of the phagocyte system, which is responsible for recognizing and degrading foreign bodies [29,30]. The main aim in different therapies is to avoid quick clearance by the body, and hence, to maintain their circulation as long as possible [31,32].

In previous works [33,34], we loaded or linked NPs to hydrogels, tuning the release taking advantage of the ratio between NPs diameter and mean mesh size. This approach could be improved because HG networks are inhomogeneous systems and physical entrapment is not sufficient. In this work, we now consider the electrostatic interactions that could take place among solutes and polymers to tune NPs release through HG.

According to the critical issues of both these polymeric systems, we considered the possibility to create a composite system of HG loaded with NPs, able to tune the release of NPs both at neutral and acidic pH. Used hydrogel was specifically developed for Central Nervous System (CNS) repair strategies and obtained from statistical block microwave-assisted polycondensation between agarose and carbomer 974P, together with cross-linkers. In previous work, we built a material library by means of components mutual ratios and hereafter they are briefly termed as AC [9,35]. We verified their high biocompatibility in vitro and in vivo and that they can remain localized at the site of injection [9], and can provide short term delivery for neuroprotective compounds and long-term for neuroregenerative ones. NPs were prepared starting from poly(ethylene glycol)-block-poly(-lactic acid) (PEG-*b*-PLA) copolymer.

In this work, we rationalize the possibility of building a composite material that could allow the release or not of NPs depending on medical needs, whilst the release of the drug is maintained the same. We focus our attention on the role of electric charges interactions between HG and NPs to tune the release rate of NPs. In defining such a composite material library, the influence of both NPs electric charge and HG mean mesh size was considered and their role was precisely understood, underlying the key role of NPs superficial charges and hydrogel mean mesh size.

This study is good proof of concept of the possibility to release or not NPs, maintaining them or not, in the target site being uptaken by phagocytes or not.

## 2. Results and Discussion

### 2.1. AC-NPs Composite Systems Synthesis

**Figure 1:**
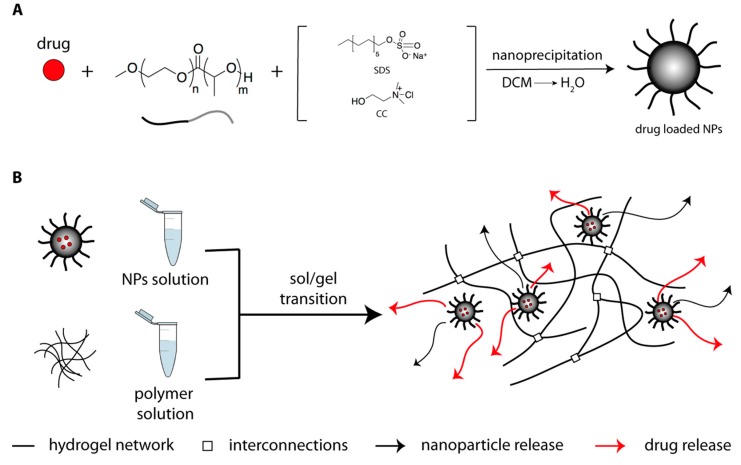
(**A**) Biodegradable core-shell nanoparticles prepared by nano-precipitation of amphiphilic poly(ethylene glycol)-block-poly(lactic acid) (PEG-*b*-PLA) copolymers, synthesized by organocatalytic ring-opening polymerization, from DCM (a good solvent for both blocks) into H_2_O (a selective solvent for the PEG block). (**B**) Schematic representation of the AC-NPs composite releasing system.

We prepared PEG-*b*-PLA using organocatalyzed ring-opening polymerization techniques, according to literature procedures (reaction scheme and NMR spectrum in Supplementary Figure S1) described in Reference [36]. This polymer was used for the preparation of PEG-*b*-PLA NPs, in a size regime suitable to AC hydrogel formation. Following preparation of PEG-*b*-PLA NPs by nanoprecipitation (Figure 1A), according to literature procedures, we introduced two different kind of surfactants to obtain differently charged NPs, i.e., choline chloride (CC) for positive charges and sodium dodecyl sulphate (SDS) for negative ones. Nanoparticles size and their size distribution, together with ζ−potential from dynamic light scattering (DLS) analysis are reported in Table 1.

NPs produced were loaded within AC6 formulation at sol state (mean mesh size 30 nm, details in Materials and Methods), before gelation to entrap them within the three-dimensional polymeric network. Heating AC sol to 70 °C leads to a higher macromer mobility with consequent short-range interconnections between the functional groups present (i.e., carboxyl groups present in carbomer 974p and hydroxyl ones present in agarose, propylene glycol, and glycerol).

During temperature decrease, polycondensation continues with a consequent increase of system viscosity and decrease of the probability of interaction between distant macromer reactive sites. This increases the possibility of an efficient reaction between closer functional groups. The result obtained at gel point (37 °C), could be schematized as a welding between the microgels surfaces that then give rise to the final three-dimensional macrostructure. By the inverted test tube all the samples gelled in less than 5 min, without any kind of differences between them. Gelation phenomenon is rapid for these systems, underlining their suitability for biomedical applications.

The obtained three-dimensional hydrogels are nanostructured and exhibit anionic nature due to the high presence of carboxylate groups. Indeed, the presence of phosphate buffered saline solution (PBS) salts freely solvated in solution increases the presence of electrolytes within the system, with consequent salt carboxylates formation [37].

### 2.2. Rhodamine Release

The in vitro drug release kinetics of Rhodamine B base (RhB), loaded in neat (a) AC6 hydrogels and in (b) NPs then loaded in AC6 hydrogels, were deeply investigated and compared.

**Figure 2:**
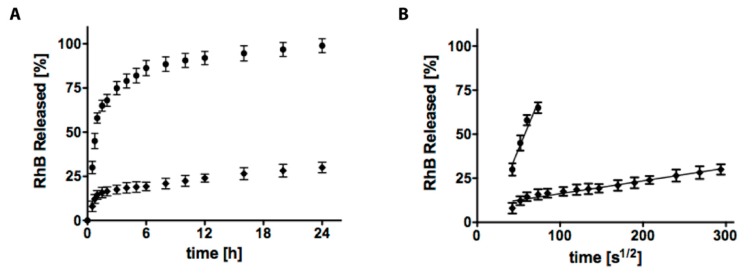
(**A**) In vitro release profiles of RhB from AC6 hydrogel (●) and AC6-NPs_SDS composite system (♦) in PBS, at 37 °C. (**B**) The slope of RhB releases against the square root time, represents how the delivery system influences Fickian diffusion.

In Figure 2, AC6 and AC6-NPs_SDS release profiles are presented (RhB release from AC6-NPs_CC shows similar trends and is presented in Supplementary Figure S2 due to clarity of the Figure). The key point of this paper is to prove that NPs can release active compounds with tailored kinetic on one side, and on the other, the mutual advantages of combining hydrogels together with NPs in a composite system. In this paper, we decided to use RhB due to the steric hindrance similar to many corticosteroids and anti-inflammatory drugs typical of pharmacological treatments. RhB was loaded within neat AC hydrogels and NPs (loading percentage = 68.5% for both NPs_CC and NPs_SDS).

RhB release profile from AC6 hydrogel samples, as visible from Figure 2A, is very rapid and completed after 12 h. RhB loaded within AC6-NPs_SDS was prolonged during time, and therefore, it represents a very promising solution. To better comment on RhB release kinetic, we plotted release percentage against time square root (Figure 2B). Here, a linear trend is typical of the Fickian diffusion mechanism and the *y*-axis intercept indicated burst release, so uncontrolled release. In the first case, (RhB loaded within AC6 hydrogel, black circle) the linear plot is visible only in the first time points, followed by a plateau trend, and a consequent high burst release contribution (about 30%). This value is typical of a system with poor ability to control the release of small steric hindrance drugs. Loading RhB within NPs and then into AC6 hydrogel is a good strategy to stretch the linear trend (Fickian diffusion as said above), and so the performance of the drug delivery system (black rhombus, Figure 2B). Here, only 5% of the drug loaded is released as burst release. The reason is that NPs can better control the release of drugs, being a more efficient barrier. Moreover, hydrogel represents another diffusion barrier that should be passed by drugs before its release. Then, once we prove that NPs can release with almost a zero-order release RhB, we will focus our attention on the possibility of building a tunable composite system able to release or not NPs contained.

### 2.3. NPs Release from AC6 Hydrogel

After having verified the better performances, in terms of drug delivery of RhB loaded within AC6-NPs network, we investigated the possibility of building a composite library taking advantage of NPs with different electric charges.

In previous works, we observed that physical obstruction could be an efficient strategy [33], but NPs being part of the polymeric network at sol state and taking part to gelation their distribution is not homogeneous, and part of them that are present at the interface gel/PBS are able to escape. Indeed, the high percentage of NPs present at the interface hydrogel/water does not allow a proper and sustained release of them. To solve these drawbacks, several research groups introduced functionalization strategies to link solutes or NPs to the HG network with cleavable bonds [34,38,39]. In these cases, release is delayed by the stability or affinity of the bond between NPs and polymer, i.e., higher the stability and slower the release kinetics. This approach is very efficient on one side, but on the other, drug loading within NPs is then more complex and most of the drug loaded is then dispersed during functionalization steps. In this work to improve the possibility of tuning the release of NPs, we considered the influence of particle electric charge on the release kinetics, i.e., positive and negative NPs were loaded within different hydrogel networks. Using this strategy, we aimed to control release rates of NPs without any chemical reaction between NPs and the HG polymeric network.

**Figure 3:**
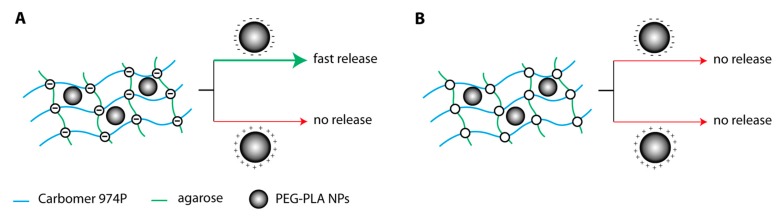
Schematic representation of NPs release from: (**A**) AC6 hydrogel at pH = 7.4; (**B**) AC6 hydrogel at pH = 5.

Figure 3 shows a schematic representation of the NPs behavior, within these polymeric matrices at pH = 7.4. As explained above, AC6 network is negatively charged at pH = 7.4, and so it is able retain positively charged NPs and release negatively charged ones (Figure 3A). Therefore, if we need to release drugs directed to macrophages, we must load them within NPs_SDS, while if we would like to avoid this uptake, it is necessary to consider NPs_CC. At pH = 5, AC6 network is neutral [40], and so NPs are not able to escape due to their diameter, which is bigger with respect to the mean mesh size of AC6 hydrogel (data on AC structural parameters in Supplementary Table S2).

**Figure 4:**
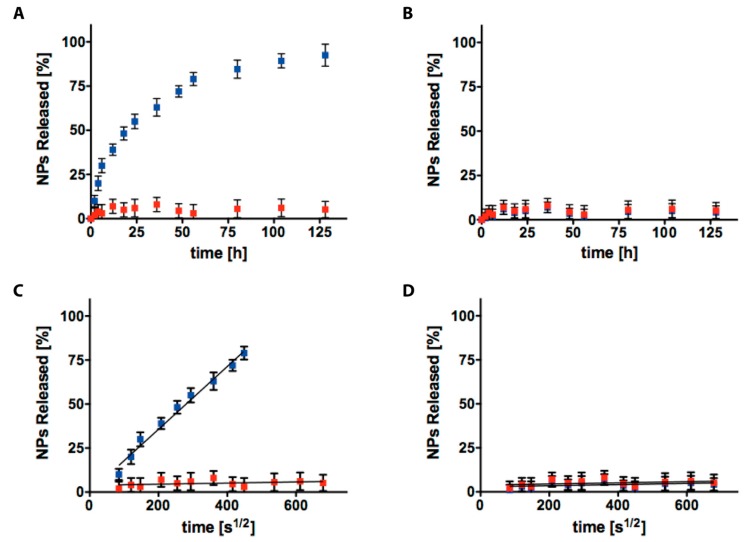
Cumulative release of NPs with different electric charges (negative, blue squares and positive, red squares) from AC6 hydrogel at pH = 7.4 (**A**) and pH = 5 (**B**); the slope of NPs released from AC6 hydrogel against the square root of time represents the role of NPs electric charges in mass transport at pH = 7.4 (**C**) and pH = 5 (**D**). The values are calculated as a percentage, with respect to the total mass loaded (mean value ± standard deviation is plotted).

In Figure 4A, NPs_SDS (blue) and NPs_CC (red) release profiles from AC6 at pH 7.4, are presented. In both cases, NPs are released through a biphasic trend characterized by: (i) an initial burst release then, (ii) followed by a slower sustained release phase which was visible for 7 days. Referring to NPs_CC, the electrostatic interactions were able to stop release rates, providing no release. Then, to investigate the differences in terms of diffusion coefficient of NPs through AC6, we plotted the cumulative percentage of release, against the time to the power of 0.5 (t^1/2^, in Figure 4C), where a linear pattern is typical of Fickian diffusion [38]. The slopes in the linear region for NPs_CC and NPs_SDS were compared, and it was well visible that the relative diffusion coefficients were very different (*p* < 0.0001). These results confirmed that the release of NPs_SDS from AC6 is still mediated by Fickian diffusion, whilst release of NPs_CC was not visible. Thus, the introduction of positive charges allowed reduction of the burst release from 20% to 2%. In Figure 4B,D, NPs_SDS (blue) and NPs_CC (red) release profiles from AC6 at pH 5, are presented. NPs characterization at pH = 5 visible in Supplementary Table S3. It was well observable that in this environment, NPs cannot escape from the AC6 matrix.

This AC6-NPs composite system, as described above in Figure 3 and Figure 4, is not able to release NPs in an acidic environment. Then, if pharmaceutical therapy needs to be released selectively within specific cells and so release of NPs is necessary, we can tune AC formulation increasing the value of the mean mesh size (Table S2) [41].

**Figure 5:**
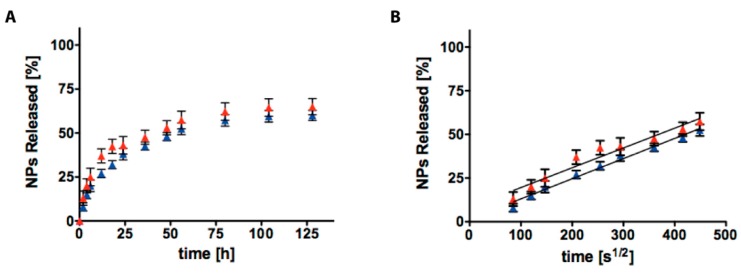
Cumulative release of NPs with different electric charges (negative, blue triangles and positive, red triangles) from AC1 hydrogel at pH = 5 (**A**); the slope of NPs released from AC1 hydrogel against the square root of time represents the role of NPs electric charges in mass transport at pH = 5 (**B**). The values are calculated as a percentage, with respect to the total mass loaded (mean value ± standard deviation is plotted).

In Figure 5, results at pH = 5 of AC1-NPs_SDS and AC1-NPs_CC release from AC1, are presented (schematic release presented in Supplementary Figure S3). In both cases, NPs are released following a biphasic trend with an initial burst release, followed by a slower release phase, which was visible for 7 days (until 14 days, data not shown).

We plotted the percentage of cumulative release of NPs through AC1, against time to the power of 0.5 (t^1/2^, in Figure 4C), to study the differences in the diffusion coefficient, where a linear relationship is indicative of Fickian diffusion. By comparing the slopes in the linear region for NPs_CC and NPs_SDS, it was observable that the relative diffusion coefficients were not different for the two cases (*p* < 0.0001). These results confirmed that the release of NPs_SDS and NPs_CC from AC1, was still mediated by Fickian diffusion and electric charges cannot influence these rates. Therefore, in this case, the introduction of positive charges does not reduce the burst release. In all the cases studied NPs released do not differ from the loaded ones (results in Supplementary Tables S4 and S5).

### 2.4. NPs Diffusivity Evaluation

Mass release data obtained experimentally (Figure 4 and Figure 5), were used to estimate NPs diffusion coefficients. As explained above, the release mechanism could be considered as a pure Fickian diffusion, being concentration driven through AC hydrogel pores [33,34]. Table 2 shows the dependence of NPs diffusivity, on their electric charges and AC mean mesh size. It was well visible that at pH = 7.4, the highest diffusion coefficient was represented by NPs-SDS loaded within AC6, while NPs_CC cannot escape from the AC6 polymeric network. To guarantee proper release rates of PEG-*b*-PLA NPs from AC hydrogels, we can tune hydrogel formulation, increasing mean mesh size following previous studies, as outlined in Reference [41]. AC1 hydrogel could allow release of NPs with different diffusivities, depending on the superficial charges of NPs, i.e., negative NPs escape quicker than positive ones, due to the mutual repulsion from negative charges of AC1 and NPs_SDS. In an acidic environment, NPs cannot diffuse through AC6 and are promising tools to avoid NPs uptake. They can diffuse through AC1 without evident differences between positively or negatively charged.

## 3. Conclusions

The main advantage behind controlled drug delivery strategies is to avoid risks due to overdosing, together with the inefficacy of underdosing, to maintain the drug level between these two boundary conditions, with consequent less amount of drug needed. In this framework, polymeric NPs are good candidates for controlled release of different drugs, but their ability is highly reduced by biodistribution and macrophage uptake, though they are needed in some medical applications.

This direction hydrogel together with drug-loaded nanoparticles could build a composite system able to guarantee proper control of release rate, avoiding the limits underlined above. In this work, we rationalized the role of electric charges interactions between HG and NPs to tune the release rate of NPs. In defining such a composite material library, the influence of both NPs electric charge and HG mean mesh size was considered, and their role was precisely understood, emphasizing the key role of NPs superficial charges, being here more influential than hydrogel mean mesh size.

## 4. Materials and Methods

### 4.1. Materials

For NPs synthesis, polyethylene glycol (PEG, 5 kDa), 1,8-diazabicycloundec-7-ene (DBU), lactic acid (LA), sodium dodecyl sulphate (SDS), and choline chloride (CC) were purchased from Sigma-Aldrich (Darmstadt, Germany) and used as received. For hydrogel synthesis, branched polyacrylic acid carbomer 974P (1MDa, 20% branched degree) was purchased from Fagron (Rotterdam, The Netherlands), phosphate buffered saline solution (PBS), propylene glycol, glycerol, and sodium hydroxide were purchased from Sigma-Aldrich (Germany), whilst agarose was obtained from Invitrogen (Carlsbad, CA, USA); and all were used as received. Rhodamine B base (RhB) (Sb sensitivity < 0.1 mg mL^−1^, Carlo Erba reagents, Cornaredo, Italy) was used for release experiments.

### 4.2. PEG-b-PLA Synthesis

PEG (0.25 g, 4.1 mmol) and DBU (10.6 mg, 10 mL, 1.0 mol% relative to LA) were dissolved in dichloromethane (DCM; 1.0 mL) [42,43]. LA (1.0 g, 6.9 mmol) was dissolved in DCM (3.0 mL) with mild heating. The LA solution was then added rapidly to the PEG/DBU solution, and was allowed to stir rapidly for 10 min. The reaction mixture was then quenched by addition of acetone (7.0 mL) and the PEG-*b*-PLA copolymer was recovered by precipitation from cold diethyl ether, collected by filtration and dried under vacuum to yield a white amorphous polymer (1.15 g, 92%). GPC (THF): M_n_ (PDI) = 25 kDa.

### 4.3. PEG-b-PLA NPs Synthesis and Drug Loading

The nanoprecipitation method was employed for the formation of PLGA–*b*–PEG NPs, as described in Reference [42]. Briefly, 100 mg of PEG-PLA were dissolved in 1 mL of DCM and dropped into the surfactant solution (0.3% *w*/*v*), while vortexing the mixture to guarantee homogenization. Surfactants used to obtain different superficial electric charges were CC (positive NPs) and SDS (negative NPs). The final mixture was sonicated three times for 10 s, and then left stirring under hood to harden for three hours. NPs can be collected through centrifugation for 15 min at 17,000× *g*. The PLGA-*b*-PEG NPs were resuspended, washed with water, and collected likewise. Final sizes and ζ-potential of NPs were determined, referring to dynamic light scattering (DLS) measurements using a Zetasizer Nano ZS from Malvern Instruments (Worcestershire, UK, the reported data are an average value of three measurements of the same sample, dissolved in distilled water).

The concentrated latex was incubated with 1 mg/mL RhB PBS solution, for one day at room temperature, under gentle magnetic stirring. The latex was then diluted to the desired concentration. Loading efficiency (% loading) was calculated based on the following equation:

(1) $$\mathrm{Drug}\;\mathrm{Loading}=\frac{\mathrm{Drug}\;\mathrm{Entrapped}\;\mathrm{in}\;\mathrm{NPs}\;}{\mathrm{Initial}\;\mathrm{Amount}\;\mathrm{of}\;\mathrm{RhB}\;\mathrm{Added}}\cdot 100$$

### 4.4. AC-NPs Synthesis

Hydrogels were prepared by batch reaction in a PBS at about 80 °C, in which a polymeric solution was achieved by stirring polymers (1% *w*/*v* carbomer 974p and 0.5% *w*/*v* agarose) into the selected solvent, adding a mixture of cross-linking agents made of propylene glycol and glycerol (along with NaOH 1 N for pH neutralization) [9,33]. The reaction pH was kept neutral. The effective gelation and reticulation were achieved by means of electromagnetic stimulation (500 W power irradiated), for 15 s per 5 mL of polymeric solution mixed with 20 mg of NPs.

The mixing reactor was kept closed to avoid any eventual loss of solvent vapors, and the gelation was then achieved in a 48 multi-well cell culture plate (0.5 mL each with a cylinder diameter of 1.1 cm), in which the gelling solution was poured during cooling. Then, the hydrogels were washed with PBS to remove the unreacted cross-linkers. Gelation time was assessed using the inverted test tube, and compared with hydrogels without NPs [44]. Gelation was achieved in less than 10 min. In particular, from a chemistry point of view, esterification between carbomer carboxyl groups and hydroxyl groups from agarose, gives rise to a three dimensional matrix. Propylene glycol and glycerol are involved in the condensation reaction, forming local interconnections through their hydroxyl groups [9], and they also play a physical role, working as thickeners. AC formulations were numbered according as described in References [9,41], and their detailed compositions are presented in Supplementary Table S1. The different amount of cross-linking agents resulted in different mesh size values: nominal 90 nm for AC1 and 30 nm for AC6 [41]. Acting on the functionality, *f* in Flory notation, of the cross-linkers it is possible to produce more or less entangled polymeric networks, and its tunable library is presented in previous work [41].

### 4.5. Rhodamine B Release from AC Hydrogels and AC-NPs Composite System

RhB was used because its steric hindrance is similar to many small steric hindrance drugs [45]. It was loaded within (i) AC hydrogel network and (ii) within NPs then loaded in AC hydrogels (AC-NPs), to compare the release ability of these two systems. (i) RhB aqueous solution (0.1 mg/mL) was mixed with AC gelling solution, above gelation to allow good solute dispersion within polymeric network; gelation took place in steel cylinders (0.5 cm^3^, *d* = 1.1 cm); (ii) NPs (loaded with RhB as explained in Section 4.3) solution was mixed with AC gelling solution, above gelation to allow good solute dispersion within polymeric network. For release studies, samples (*n* = 3) were loaded within a dialysis bag (cut-off = 1000 Da) and put in excess of PBS, and aliquots were collected at defined time points, and the sample volume replaced by fresh PBS, in order to avoid mass-transfer equilibrium between the gel and the surrounding solution. Percentage of RhB released was measured by UV spectroscopy [46].

### 4.6. NPs Release from AC Hydrogels and Mathematical Modeling

AC1 and AC6 hydrogels were loaded with different PEG-*b*-PLA NPs, to assess their in vitro diffusion-controlled delivery. Composite materials were briefly named as follows: AC1-NPs_CC for AC1 hydrogel loaded with positive NPs, etc. NPs (10 mg/mL) were loaded as RhB during the cooling phase of AC hydrogels during sol state, i.e., before gelation [9].

AC loaded with NPs (AC-NPs) after gelation were put in a NPs isotonic solution within a standard incubator at 37 °C and 5% CO_2_ atmosphere, overnight, to allow swelling without having any NPs release [41].

Hence, as characteristic time is much larger than experimental length, degradation reactions can be taken as fully neglectable in this framework. Therefore, NPs flux was controlled only by concentration gradient and diffusion can be considered as Fickian.

Hydrogels were then placed in excess of PBS (pH = 7.4) and water at pH = 5 filled wells (5 mL volume each). Aliquots were collected at defined time points, and the sample volume replaced by fresh solution. Samples collected were analyzed to assess the cumulative NPs release percentage using DLS [33]. In addition, to fully recapitulate this AC-NPs composite material library, NPs diffusivity (D) was evaluated with a mathematical model based on mass balances, i.e., on fundamental conservation laws [47,48]. Diffusion is described through the second Fick law, with a 1-dimensional model in a cylindrical geometry, as mathematical details are presented in previous work, Reference [31].

### 4.7. Statistical Analysis

Where applicable, experimental data were analyzed using Analysis of Variance (ANOVA). Statistical significance was set to *p* value < 0.05. Results were presented as mean value ± standard deviation.

## Figures and Tables

**Table 1:** Characteristics of the produced nanoparticles (NPs) as measured by dynamic light scattering (DLS). Polydispersity index (PDI); sodium dodecyl sulphate (SDS); choline chloride (CC).

	Diameter (nm)	PDI (–)	ζ−Potential (mV)
**NPs_SDS**	75.4	0.12	−21.5
**NPs_CC**	81.2	0.15	28.5

**Table 2:** Diffusion coefficients (D) of NPs_SDS and NPs_CC from AC6 and AC1 hydrogels at pH = 7.4 and pH = 5.

	D ^1^ (cm^2^/s)	
	pH = 7.4	pH = 5
AC6-NPs_SDS	2.99 ± 0.15	-
AC6-NPs_CC	-	-
AC1-NPs_SDS	2.25 ± 0.18	1.34 ± 0.12
AC1-NPs_CC	1.16 ± 0.1	1.51 ± 0.16

^1^ All values have to be multiplied by 10^−6^.

## Supplementary Materials

The following are available online at http://www.mdpi.com/2310-2861/4/3/74/s1, Figure S1: (A) PEG-*b*-PLA (**3**) ring-opening organocatalyzed synthesis from PEG (**1**) and LA (**2**). (B) NMR spectrum of PEG-*b*-PLA (**3**): characteristic peaks of the obtained product are highlighted; Figure S2: In vitro release profiles of RhB from AC6 hydrogel (black square), AC6-NPs_CC (red square) composite system in PBS at 37 °C; Figure S3: Schematic representation of NPs release from AC1 hydrogel at pH = 5; Table S1: Chemicals proportions and weights for the two AC hydrogels formulations tested; Table S2: AC hydrogel structural parameters; Table S3: characteristics of the produced nanoparticles (NPs) as measured by dynamic light scattering (DLS) at pH = 5. Table S4: Characteristics of the produced NPs, as measured by DLS after being released from AC1-NPs composite system. Table S5: Characteristics of the produced NPs, as measured by DLS after being released from AC6-NPs composite system.

## Symbols

*C*_S_	drug concentration in solution	g mL^−1^
*C*_G_	drug concentration within hydrogel	g mL^−1^
*C*_G,0_	drug concentration within hydrogel at time *t* = 0	g mL^−1^
*D*	diffusion coefficient	m^2^ s^−1^
*k_C_*	mass transfer coefficient	m s^−1^
*m*_G,0_	drug mass present inside matrix	g
*r*	hydrogel radius	cm
*S*_exc_	exchange interfacial surface	cm^2^
*Sh*	Sherwood number	-
*t*	time	s
*V*_G_	volume of hydrogel in hydrated state	cm^3^
*V*_S_	volume of solution	cm^3^
