# Supplementary material for: Hydrogel-Nanoparticles Composite System for Controlled Drug Delivery
Source: Gels. 2018 Sep 4;4(3):74. doi: 10.3390/gels4030074 (PMC6209253; doi:10.3390/gels4030074)
Supplement: Supplementary file 1 [file gels-04-00074-s001.pdf]

Supporting information

Hydrogel-nanoparticles composite system for controlled drug delivery

Emanuele Mauri [1], Anna Negri [1], Erica Rebellato [1], Maurizio Masi [1], Giuseppe Perale [2] and Filippo Rossi [1,*]

[1] Department of Chemistry, Materials and Chemical Engineering "G. Natta", Politecnico di Milano, via Mancinelli 7, 20131 Milan, Italy; E.M. emanuele.mauri@polimi.it; A.N. anna1.negri@mail.polimi.it; E.R. erica.rebellato@mail.polimi.it; M.M. maurizio.masi@polimi.it; F.R. filippo.rossi@polimi.it;

[2] Biomaterials Laboratory, Institute for Mechanical Engineering and Materials Technology, SUPSI – University of Applied Sciences and Arts of Southern Switzerland. Via Cantonale 2C, Galleria 2, 6928 Manno, Switzerland; G.P. giuseppe.perale@supsi.ch;

* Correspondence: filippo.rossi@polimi.it; Tel.: +39-02-2399-3145.

PEG-b-PLA synthesis and characterization

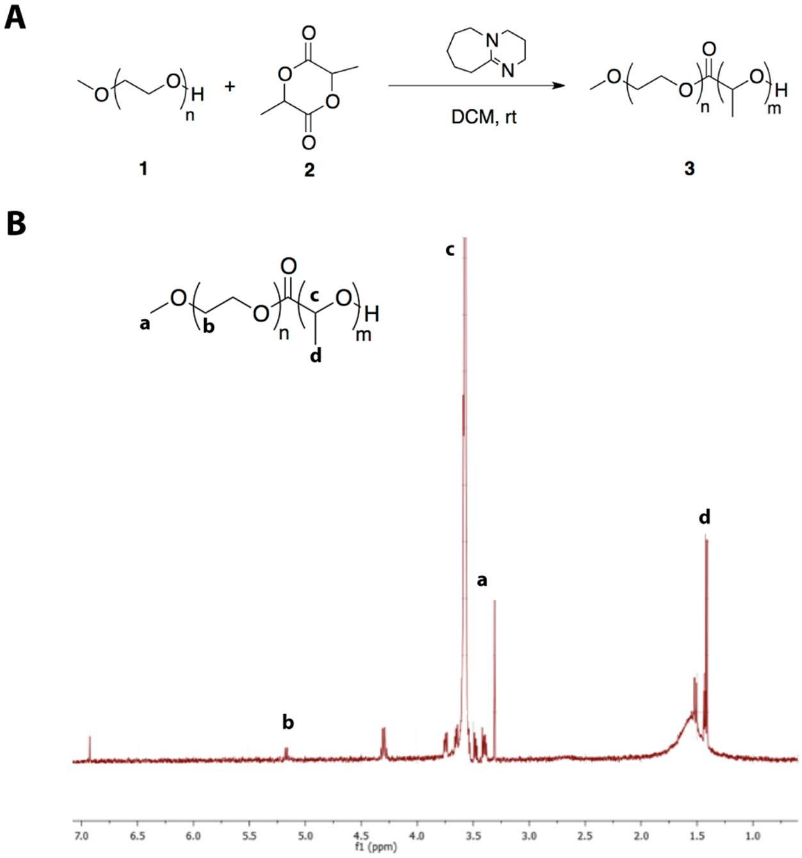

Figure S1. (A) PEG-b-PLA (**3**) ring-opening organocatalyzed synthesis from PEG (**1**) and LA (**2**). (B) NMR spectrum of PEG-b-PLA polymer (**3**): characteristic peaks of the obtained product are highlighted.

PEG-b-PLA synthesis and characterization

In the present study, two different hydrogel formulations were tested, varying the number of constituents used (AC1 and AC6). Table S1 shows proportions (weight percentages) used in this work. Acronyms of hydrogels were harmonized with previous studies.

Table S1. Chemical proportions and weights of the two AC hydrogel formulations tested.

HG component	AC1	AC6
PBS [mL]	100	69
carbomer 974P [g]	0.5	0.5
agarose [g]	0.5	0.5
propylene glycol [mL]	0	30
glycerol [mL]	0	1

AC-NPs composite systems synthesis

AC hydrogel structural parameters were calculated using Flory-Rehner theory and presented in previous work [41]; also presented in Table S2.

Table S2. AC hydrogel structural parameters.

	mean mesh size [nm]
**AC6 pH=7.4**	30
**AC6 pH=5**	25
**AC1 pH=7.4**	90
**AC1 pH=5**	85

AC-NPs composite systems synthesis

NPs diameter and ζ-potential at pH=5 were measured using DLS (Table S3).

Table S3. Characteristics of the produced nanoparticles (NPs) as measured by dynamic light scattering (DLS) at pH=5.

	diameter [nm]	PDI [-]	ζ-potential [mV]
**NPs_SDS**	72.4	0.15	-3.35
**NPs_CC**	102.6	0.2	34.5

Rhodamine release

RhB release from AC6 and AC6-NPs_CC is presented in Figure S2.

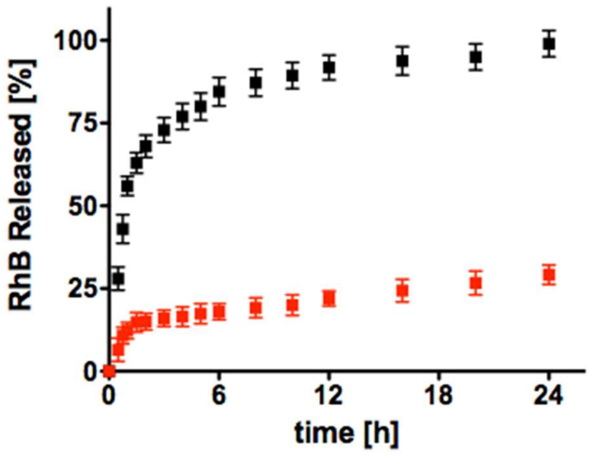

Figure S2. In vitro release profiles of RhB from AC6 hydrogel (b), AC6-NPs_CC (red square) composite system in PBS at 37 °C.

NPs release from AC1 hydrogel

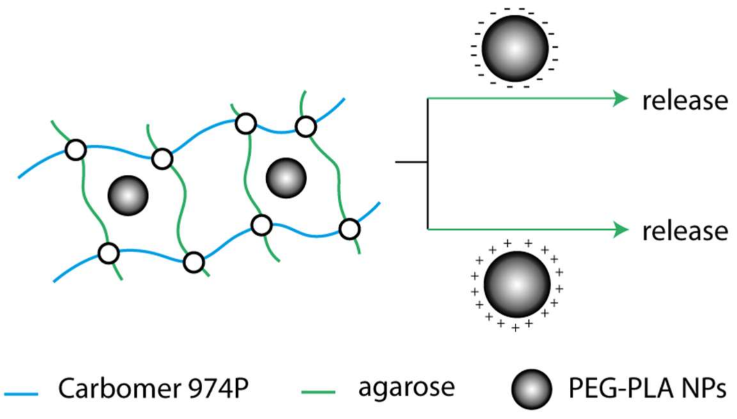

Figure S3. Schematic representation of NPs release from AC1 hydrogel at pH = 5.

NPs characterization after being released from AC-NPs composite system

NPs diameter and ζ-potential after being released from AC1-NPs were measured using DLS (Table S4).

Table S4. Characteristics of the produced NPs as measured by DLS after being released from AC1-NPs composite system.

	**diameter [nm]**	**PDI [-]**	**ζ-potential [mV]**
**NPs_SDS (pH=7.4)**	74.8	0.14	-19.6
**NPs_CC (pH=7.4)**	83.5	0.16	27.8
**NPs_SDS (pH=5)**	73.4	0.16	-3.4
**NPs_CC (pH=5)**	104.6	0.18	29.8

NPs diameter and ζ-potential after being released from AC6-NPs were measured using DLS (Table S5).

Table S5. Characteristics of the produced NPs as measured by DLS after being released from AC6-NPs composite system.

	**diameter [nm]**	**PDI [-]**	**ζ-potential [mV]**
**NPs_SDS (pH=7.4)**	76.9	0.13	-23.6
**NPs_CC (pH=7.4)**	82.4	0.17	26.5
**NPs_SDS (pH=5)**	71.4	0.17	-3.56
**NPs_CC (pH=5)**	99.8	0.21	32.3

References

41. Rossi, F.; Perale, G.; Storti, G.; Masi, M. A library of tunable agarose carbomer-based hydrogels for tissue engineering applications: The role of cross-linkers. *J. Appl. Polym. Sci.* **2012**, *123*, 2211-2221.

© 2018 by the authors. Submitted for possible open access publication under the terms and conditions of the Creative Commons Attribution (CC BY) license (http://creativecommons.org/licenses/by/4.0/).
